# Environmental monitoring of SARS-CoV-2 in the metropolitan area of Porto Alegre, Rio Grande do Sul (RS), Brazil

**DOI:** 10.1007/s11356-023-31081-8

**Published:** 2023-12-06

**Authors:** Leticia Batista Dutra, Janaína Francieli Stein, Bruna Seixas da Rocha, Andresa Berger, Beatriz Andrade de Souza, Bruno Aschidamini Prandi, Arthur Tonietto Mangini, André Jarenkow, Aline Alves Scarpellini Campos, Fernando Mainardi Fan, Maria Cristina de Almeida Silva, Katia Helena Lipp-Nissinen, Manuel Rodrigues Loncan, Matheus Ribeiro Augusto, Ana Cláudia Franco, Rodrigo de Freitas Bueno, Caroline Rigotto

**Affiliations:** 1 Laboratory of Molecular Microbiology and Cytotoxicity, Health Sciences Institute, Feevale University, ERS 239 n° 2755, Novo Hamburgo, RS CEP 93352-000 Brazil; 2 Division of Laboratories, Henrique Luis Roessler State Foundation for Environmental Protection (FEPAM), Porto Alegre, RS CEP 90020-021 Brazil; 3 Virology Laboratory, Institute for Basic Health Sciences, Federal University of Rio Grande do Sul, Porto Alegre, RS CEP 90050-170 Brazil; 4 State Center for Health Surveillance, Rio Grande do Sul State Health Department, Porto Alegre, RS CEP 90119-900 Brazil; 5 Hydraulic Research Institute, Federal University of Rio Grande do Sul, Porto Alegre, RS CEP 91501-970 Brazil; 6 Center of Engineering, Modelling and Applied Social Sciences (CECS), Federal University of ABC (UFABC), Santo Andre, SP CEP 09210-580 Brazil

**Keywords:** Coronavirus, Sewage, Surface water, COVID-19, WBE

## Abstract

Since starts the coronavirus disease 2019 (COVID-19) pandemic identified the presence of genomic fragments of *severe acute respiratory syndrome coronavirus 2* (SARS-CoV-2) in various environmental matrices: domestic sewage, surface waters, and contaminated freshwater. Environmental monitoring of SARS-CoV-2 is a tool for evaluating trend curves over the months, compared to several clinical cases of the disease. The objective of this study was to monitor the SARS-CoV-2 in environmental samples collected in different sites in a metropolitan area of Porto Alegre, Southern Brazil. During 10 months from 2020 to 2021, 300 samples were collected weekly and biweekly from nine points located in 3 cities: one point from a wastewater treatment plant (WWTP) in São Leopoldo (fortnightly collection), two points in Dilúvio Stream in Porto Alegre (fortnightly collection), two points in Pampa and Luiz Rau Streams (weekly collection), and two points in public fountains (fortnightly collection) in Novo Hamburgo. After collection, samples were concentrated by ultracentrifugation, and viral nucleic acids were extracted using MagMax® Core Nucleic Acid Purifications kits and submitted to RT-qPCR, using E, N1, and N2 gene targets of SARS-CoV-2. Only 7% (3/41) samples from public fountains were positive, with a mean viral load (VL) of SARS-CoV-2 RNA of 5.02 × 10^1^ gc/l (2.41~8.59 × 10^1^ gc/l), while the streams had average VL of 7.43 × 10^5^ gc/l (Pampa), 7.06 × 10^5^ gc/l (Luiz Rau), 2.01 × 10^5^ gc/l (Dilúvio), and 4.46 × 10^5^ cg/l (WWTP). The results showed varying levels of viral presence in different sample types, with a demonstrated correlation between environmental viral load and clinical COVID-19 cases. These findings contribute to understanding virus persistence and transmission pathways in the environment. Continuous monitoring, especially in less developed regions, is crucial for early detection of vaccine resistance, new variants, and potential COVID-19 resurgence.

**Supplementary Information:**

The online version contains supplementary material available at 10.1007/s11356-023-31081-8.

## Introduction

The World Health Organization (WHO) declared the first human case of coronavirus disease 2019 (COVID-19), a coronavirus disease caused by *severe acute respiratory syndrome coronavirus 2* (SARS-CoV-2), in March 2020, reported in December 2019 from Wuhan City, China (WHO 2021). Since the announcement, many patients have fallen ill, and there have been casualties worldwide. Human transmission of SARS-CoV-2 is primarily airborne (Zhang and Ma 2020). However, numerous investigations have demonstrated the presence of SARS-CoV-2 in faeces (Gu et al. 2020; Guan et al. 2020; Wang et al. 2020; Wu et al. 2021; Xiao et al. 2020; Yuen et al. 2020), even before the development of clinical symptoms (Duvallet et al. 2022).

The COVID-19 pandemic has sparked a significant increase in interest in the use of wastewater-based epidemiology (WBE) as a complement to clinical testing data. Clinical testing can be limited due to factors such as test-seeking behaviour and availability (Wu et al. 2020). Recent empirical and inferential evidence suggests that environmental surveillance in low-income countries (LICs) should expand to include four additional components: raw/untreated wastewater/effluents from on-site sanitation systems, raw/untreated surface water and groundwater, drinking water systems, and solid waste. This expansion of traditional WBE forms the basis of the novel wastewater, waste, and water-based epidemiology (WWWBE) hypothesis and decision-support tool (Gwenzi 2022).

Studies from several countries have reported the presence of the novel coronavirus in wastewater, including the Netherlands (Medema et al. 2020), USA (Duvallet et al. 2022; Sherchan et al. 2020; Toledo et al. 2022), UK (Wade et al. 2022), South Africa (Tlhagale et al. 2022), Japan (Tanimoto et al. 2022; Zhu et al. 2022), France (Foladori et al. 2020; Wurtzer et al. 2020), Italy (Rimoldi et al. 2020a, 2020b; Forthomme  2020), Spain (Chavarria-Miró et al. 2021; Randazzo et al. 2020), India (Kumar et al. 2022a, 2021, 2020), Brazil (Ayrimoraes et al. n.d.; Chernicharo et al. 2021a; Claro et al. 2021; Fongaro et al. 2021a, 2021b; Pepe Razzolini et al. 2021; Prado et al. 2020; Salvato et al. 2021), and Australia (Ahmed et al. 2021, 2020), and in surface water or underground water: Iran (Jeddi et al. 2022), Mexico (Mahlknecht et al. 2021; Rosiles-González et al. 2021; Zarza et al. 2022), India (Kumar et al. 2022a, 2022b, 2021, 2020), Ecuador (Guerrero-Latorre et al. 2020a), Brazil (Fongaro et al. 2021a; Pepe Razzolini et al. 2021), Serbia (Kolarević et al. 2021), and Dominican Republic (Rodríguez Rodríguez et al. 2021). Many of these studies focused their analyses on sampling sewage at treatment plant influents or at intermediate locations, such as sewage pumping stations, since those points concentrate the wastewater of the monitored population. However, especially in low-income regions, there is a lack of coverage in sewage collection and treatment systems, leading to waste disposal in water bodies such as rivers, streams, and oceans near urban areas.

From the innovative perspective of monitoring urban streams and the need to comprehend the environmental circulation of SARS-CoV-2 in less developed countries with limited sewage treatment coverage, this study aimed to evaluate urban streams, public water fountains, and wastewater treatment plants (WWTP) in municipalities of RS, the southernmost state in Brazil, from October 2020 to August 2021.

## Materials and methods

### Study area

Rio Grande do Sul (RS) was one of the Brazilian states least affected during the first wave of the COVID-19 pandemic (from early July to September 2020). However, cases increased sharply from early November to late December 2020 (SES 2021a). From May to October 2020, it was observed that the most common strains were B.1.1.33 and P.2 (initially named B.1.1.248) (Franceschi et al. 2021). The P.2 strain, first detected in October 2020, had gradually replaced them as the predominant variant by January 31, 2021 (SES 2021b). The second pandemic wave began in Brazil only in December 2020, coinciding with the emergence of the VOC P.1.

The monitoring efforts were carried out in Porto Alegre, the capital city of RS, and two municipalities in the same metropolitan region: Novo Hamburgo and São Leopoldo. In this region, an estimated 55% of the population lacks sewage collection. The rate of sewage treatment relative to water consumption is approximately 37%, according to the National Sanitation Information System (SNIS 2020).

Porto Alegre City has a population of 1,488,252 and a sewage collection and treatment rate of 64% (SNIS 2020). Novo Hamburgo, with 247,032 inhabitants, has only 7.17% of its sewage collected and treated. São Leopoldo, located in the metropolitan region of Porto Alegre, has 238,648 inhabitants, and only 12.45% of its sewage is collected and treated (IBGE 2020).

### Sampling sites and field methods

Samples from Dilúvio Stream and Vicentina WWTP were analysed in the Virology Laboratory of the Institute of Basic Health Sciences at the Federal University of Rio Grande do Sul (UFRGS). Samples from the Pampa Stream, Luiz Rau Stream, and public water fountains were analysed in the Laboratory of Molecular Microbiology and Cytotoxicity at the Health Sciences Institute of Feevale University.

#### Field methods of sampling: Dilúvio Stream and Vicentina WWTP

These samples were collected at different sites: Dilúvio Stream Point One (DS1), Dilúvio Stream Point Two (DS2), and Vicentina Wastewater Treatment Plant (WWTP). We collected composite samples of 1000 ml each. In DS1 and DS2, composite samples were obtained manually over 4 h, with samples taken at 30-min intervals. These samples were then poured into a refrigerated container to maintain a temperature of 4±2°C throughout the collection period. During sampling at WWTP, an automatic composite sample was collected over 6 h, with the temperature maintained at 4 °C. All sampling points were registered for both sample temperature and environmental temperature.

After collection, the samples were transported to the lab at 4 °C and kept at the same temperature until processing. The data related to these points are identified in Table S1 (Supplementary Material).

#### Field methods of sampling: Luiz Rau and Pampa Stream and Water Public Fountains

These samples were collected at different sites: Luiz Rau Stream Point One (LRS1), Luiz Rau Stream Point Two (LRS2), Pampa Stream Point One (PS1), Pampa Stream Point Two (PS2), Centro Water Public Fountain (PF1), and Canudos Water Public Fountain (PF2). We collected 500-ml grab samples at these points and recorded both sample temperature and environmental temperature. The data related to these points are identified in Table S1 (Supplementary Material).

#### Sampling sites

**Fig. 1:**
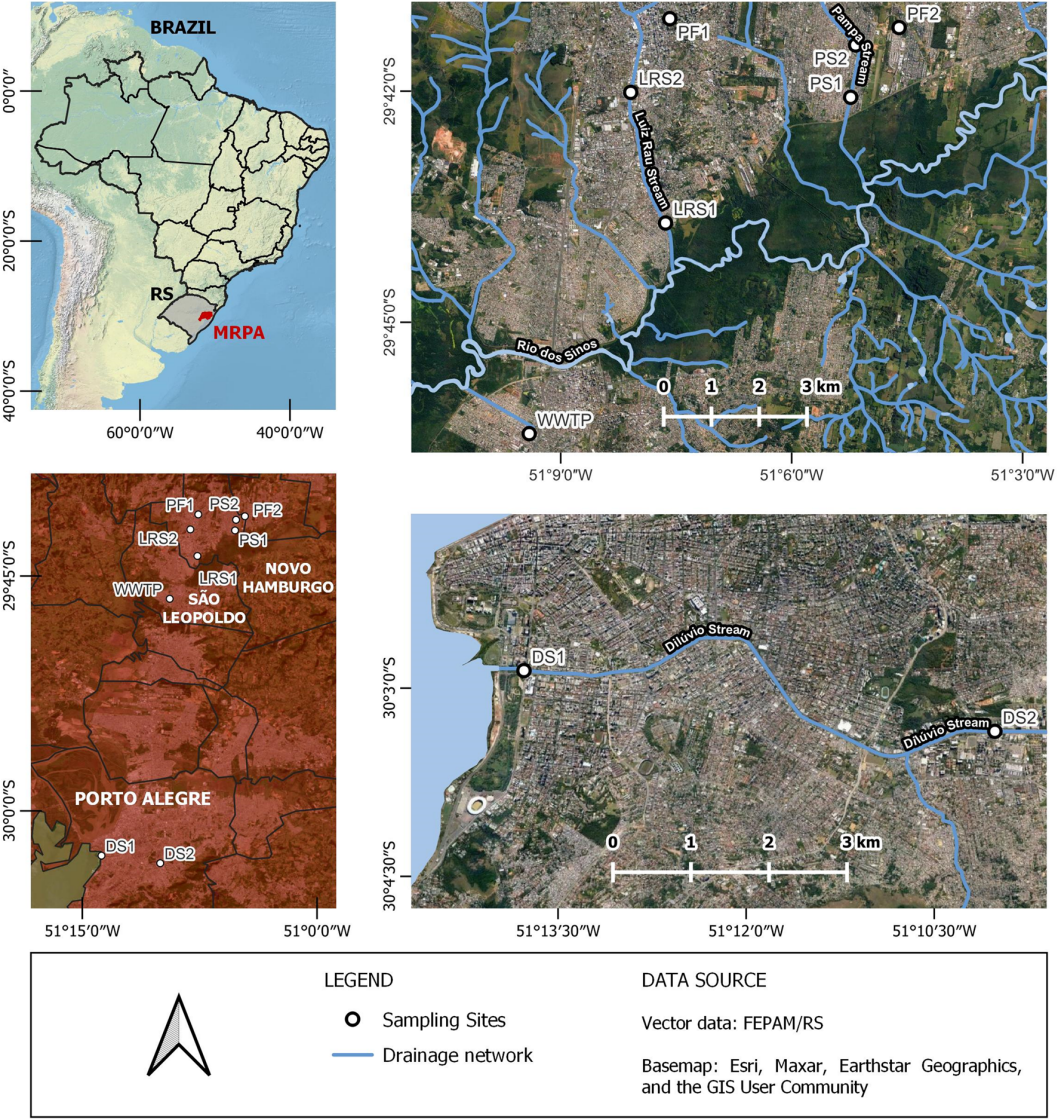
The study area and collection points are depicted in the images. In the upper left image, you can see the metropolitan area of Porto Alegre in RS, Brazil. The bottom left image shows all the sampling points. The upper right image displays Pampa Stream points one and two (PS1/PS2), Luiz Rau Stream points one and two (LRS1/LRS2), two public fountains (PF1/PF2) in Novo Hamburgo, and São Leopoldo’s Vicentina Wastewater Treatment Plant (WWTP) point. The bottom right image shows Dilúvio Stream points one and two (DS1/DS2) in Porto Alegre. Source: Petry et al. 2016

This study was conducted in three urban streams (see Fig. 1): one located in the city of Porto Alegre (Dilúvio Stream) and two in Novo Hamburgo (Pampa and Luiz Rau Streams). The streams in these regions are heavily impacted by domestic sewage discharged without any kind of prior treatment. The domestic sewage mixes with the surface waters of the streams, often making them unsuitable for human consumption. Additionally, in São Leopoldo, there was a sampling point at the Vicentina Wastewater Treatment Plant (WWTP). Novo Hamburgo also provided water samples from public fountains. The locations of these points are indicated in Fig. 1.

Physicochemical measurements, including sample temperature and environmental temperature, were conducted at all points of the Pampa Stream, Luiz Rau Stream, and public fountains using a digital thermometer (JProlab). The occurrence of rainfall was recorded on the day of collection as well as in the previous 48 h, and the presence of wind at the collection time was also noted.

All samples were labelled, refrigerated, transported to the laboratory, and stored at 4 °C until processing within 24 h.

### Laboratory methods

This work was conducted by an inter-institutional research group established shortly after the onset of the pandemic. Consequently, certain laboratory procedures were carried out based on the availability of supplies and equipment at each institution.

#### Sample concentration: Dilúvio Stream and Vicentina WWTP

The concentration process was based on ultracentrifugation (Girardi et al. 2018; Pina et al. 1998). Briefly, samples of surface water with wastewater^1^ (36 ml) were ultracentrifuged at 110,000 × g at 4 °C for 1 h. After that, the pellet was resuspended in 4 ml of Glycine 1 N pH 9.5 and then incubated for 30 min in ice with vortex agitation. Four ml of 2 X phosphate buffer saline (PBS 2X, pH 7.4) was added, and then samples were clarified at 4000 × g for 20 min to remove in-suspension particles. The supernatants were then collected and ultracentrifuged at 110,000 × g for 1 h. The supernatants were discarded, the pellet was resuspended in 500 μl of PBS 1 X buffer, and the samples were stored at −80 °C until the RNA extraction.

#### Sample concentration: Luiz Rau and Pampa Streams and Water Public Fountains

The concentration process was based on ultracentrifugation (Girardi et al. 2018) with some adaptations. In brief, water samples (36 ml) were ultracentrifuged at 41,000 × g for 3 h at 4 °C. Subsequently, the supernatant was discarded, and the pellet was eluted in 3 ml of MEM (Minimum Essential Medium) at pH 8.0, followed by vigorous homogenization through vortexing for 1 min. Afterward, aliquots were prepared in microtubes and stored in the ultra-freezer at −80 °C until further analysis.

#### RNA extraction and detection methods

For DS1, DS2, and WWTP/VSL, RNA was extracted from a concentrated sample (200 μl of eluted sample) using the Maxwell® 16 Viral Total Nucleic Acid Purification Kit with the automatic extractor Maxwell® RSC (Promega). Samples LRS1, LRS2, PS1, PS2, PF1, and PF2 were extracted using the commercial MagMax® Core Nucleic Acid Purifications kit (Thermo Fisher Scientific), following the manufacturer’s instructions. The purified nucleic acids were stored in an ultra-freezer at −80 °C.

The RT-qPCR reaction was carried out with TaqPath One-Step RT-qPCR (Thermo Fisher Scientific), and the thermocycling conditions included as follows: 15 min at 50 °C for the reverse transcription reaction, pre-heating at 95 °C for 10 min, followed by 45 amplification cycles of 15 s at 95 °C and 30 s at 55 °C. Reactions were considered positive when the cycle threshold was below 40 cycles. Positive samples were quantified using a standard curve created with serial dilutions (1:10) from the SARS-CoV-2-positive control with a known genomic copy concentration previously quantified in digital droplet PCR (ddPCR) (Monteiro and Santos 2017). The standard curve was generated using a positive control quantified by digital PCR. The detection limit considered was 5 genomic copies (gc)/μl (5.0 × 10^−6^ gc/l). Each qPCR run included SARS-CoV-2 RNA-positive controls of different known concentrations and a negative control.

In this study, we evaluated three gene targets of SARS-CoV-2 (E, N1, and N2), except in the Dilúvio Stream and Vicentina WWTP samples, where only the N1 and N2 target genes were assessed. RT-PCR was performed using primers and probe sets previously published in the CDC_2019_N1N2 SARSCoV-2 Center for Disease Control and Prevention protocol (CDC 2020) (Table 1), which target a region of the nucleocapsid (N1 and N2) gene. In the protocol adopted by the World Health Organization (WHO) (Corman et al. 2020), the E gene targets the envelope protein (Table 1).

**Table 1:** Primer and probe assay sequences for the real-time RT-PCR panel according to the CDC (2020) and Corman et al. (2020) study for the detection of SARS-CoV-2

Assay	Genome target	Genome location	Primers and probes	Sequence, 5′→3′	Amplicons size (bp)
N1	Nucleocapsid gene	28303–28322	Forward primer	GACCCCAAAATCAGCGAAAT	73
		28374–28351	Reverse primer	TCTGGTTACTGCCAGTTGAATCTG	
		28325–28348	Probe	ACCCCGCATTACGTTTGGTGGACC	
N2	Nucleocapsid gene	29180–29199	Forward primer	TTACAAACATTGGCCGCAAA	67
		29246–29228	Reverse primer	GCGCGACATTCCGAAGAA	
		29204–29226	Probe	ACAATTTGCCCCCAGCGCTTCAG	
Assay	Genome target	Oligonucleotide		Sequence, 5′→3′	Concentration
E	Envelope	E_Sarbeco_F		ACAGGTACGTTAATAGTTAATAGCGT	Use 400 nM per reaction
		E_Sarbeco_P_1_		FAM-ACACTAGCCATCCTTACTGCGCTTCG-BBQ	Use 400 nM per reaction
		E_Sarbeco_R		ATATTGCAGCAGTACGCACACA	Use 400 nM per reaction

### Calculation of cumulative COVID-19 cases

The number of COVID-19 cases was obtained from the official COVID-19 website of the RS state government (SES-RS), specifically from the Coronavirus Panel (https://ti.saude.rs.gov.br/covid19/). This platform provides individual patient notifications with a wealth of information, including details such as the municipality, neighbourhoods, date of symptom onset, and the type of test used for confirmation, among other data not utilized in this study.

For this study, we adopted a simplified model to estimate the population contributing to cloacal discharges into the stream. We counted the clinical cases of COVID-19 from the likely neighbourhoods in Porto Alegre and Viamão, both of which are part of the Dilúvio Stream basin. In this analysis, data from asymptomatic patients and those who could not report the first day of symptoms were not included. We calculated the number of cumulative cases from the onset of illness, considering the dynamics of SARS-CoV-2 viral shedding in stool samples (Fongaro et al. 2021b; Medema et al. 2020). Therefore, our model accounts for the cumulative sum of cases within a 14-day period before the onset of symptoms, as patients typically begin shedding SARS-CoV-2 in their faeces about 14 days before showing symptoms. Additionally, we considered the delay in reporting cases to local health authorities and local factors (Barbosa et al. 2022; Claro et al. 2021; Guerrero-Latorre et al. 2020a), including the lack of proper sanitary sewage treatment.

### Statistical analysis

For all analyses, the data were tested for normality using the Shapiro-Wilk test. The choice of this test was based on the results of the preliminary analysis. We employed Friedman’s test to assess whether there was a statistical difference in viral load among the various sampling points within each stream (*n*=44). The Friedman test was similarly used to assess differences between the streams, considering the points as additive, with the samples from both points treated as replicates (*n*=88).

To evaluate potential statistical differences in viral load between the Centro Water Public Fountain (*n*=18) and the Canudos Water Public Fountain (*n*=23), we used the Mann-Whitney test.

We employed a Spearman correlation test to investigate whether there was any interference between the sample and environmental temperatures in the viral load results at the different points. This test aimed to determine whether there was an association between temperatures (environmental and water) and the obtained viral load values.

To understand whether viral load increased with the number of clinical cases, we utilized a cubic polynomial regression. We chose this test because the variables do not exhibit a linear relationship due to biological functions having an optimal temperature, which does not follow a linear pattern.

For all hypothesis tests, a significance level of 5% was applied. The statistical analysis was conducted using the Statistical Program PAST 4.11 - version 06/2022 (Hammer et al. 2001).

## Results

Over a 10-month period, from October 2020 to August 2021, we collected a total of 300 samples. They were collected weekly from the Luiz Rau and Pampa Streams and taken fortnightly from Dilúvio Stream, the Public Fountains, and the Vicentina WWTP. The main outcomes of the study are detailed below.

### Cumulative cases

To determine the cumulative cases, we searched for clinical cases of COVID-19 in each neighbourhood using the official platform provided by the RS State Government. We specifically looked for the number of daily cases confirmed by RT-qPCR. Once we obtained the daily case numbers, we calculated the sum of cases over 14 consecutive days (a moving sum) before the onset of illness symptoms. We then compared these data with the viral load (VL) results from our research.

### SARS-CoV-2 RNA in streams

In Luiz Rau Stream, among the 88 samples collected from each point (LRS1 and LRS2), the average viral load at point one was 6.52 × 10^5^ gc/l, while at point two, it was 9.66 × 10^5^ gc/l. The overall mean for both points was 8.35 × 10^5^ gc/l, with a minimum of 4.65 × 10^3^ gc/l and a maximum of 5.19 × 10^6^ gc/l.

In Pampa Stream, we collected 88 samples from each site (PS1 and PS2). The mean viral load at site one was 7.43 × 10^5^ gc/l, and at site two, it was 6.69 × 10^5^ gc/l. The combined mean for both sites was 7.06 × 10^5^ gc/l, with a minimum of 4.35 × 10^3^ gc/l and a maximum of 5.16 × 10^6^ gc/l.

**Fig. 2:**
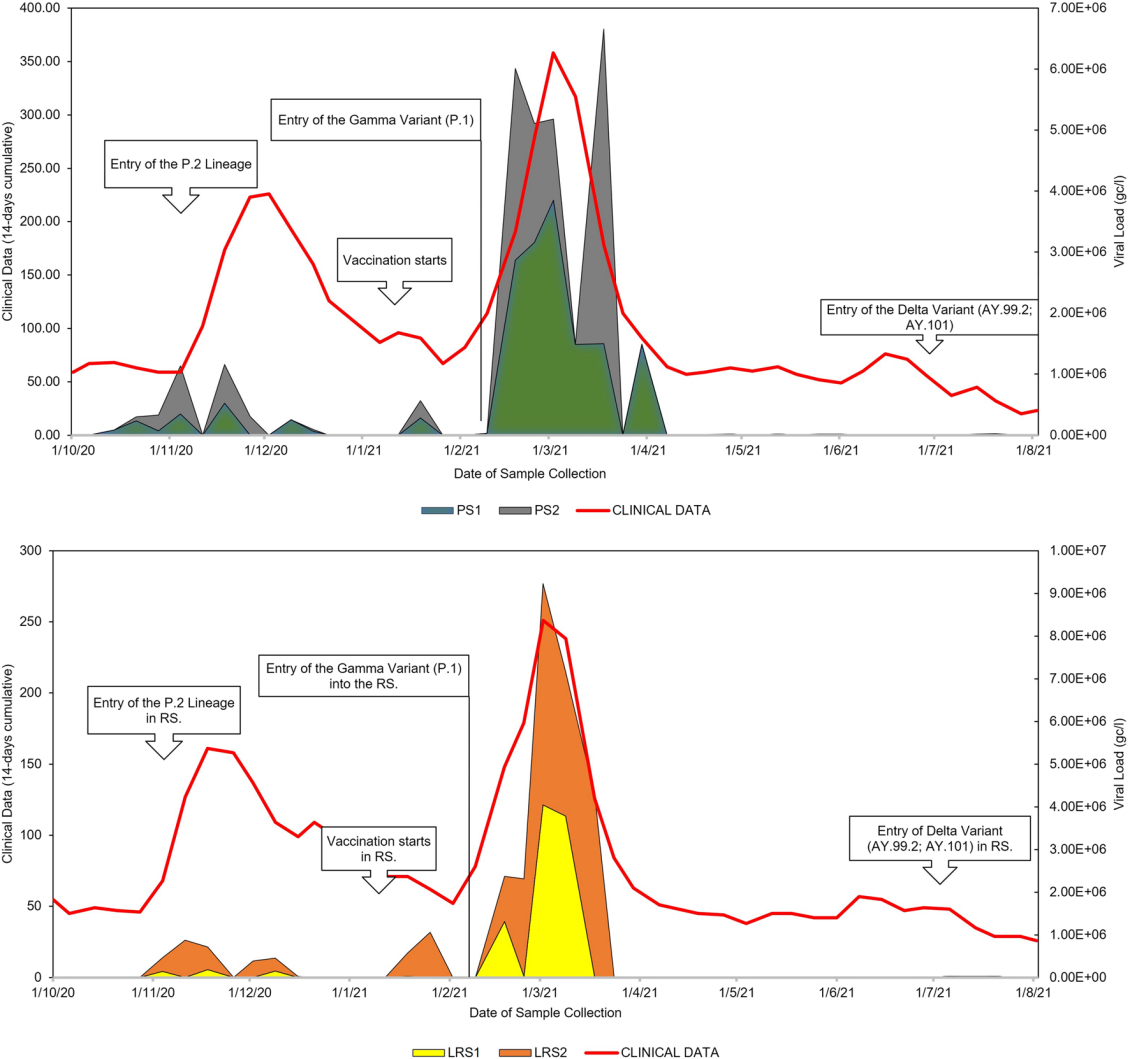
Relationship between clinical cases (14-day moving sum) and VL results: **a** in the two Pampa Stream points (in gc/l) and **b** in the two Luiz Rau Stream points (in gc/l). Source: Petry et al. 2016

In Fig. 2a, the results obtained from Pampa Stream align with the dynamics of clinical cases recorded in the study regions. The graph displays data related to clinical cases because this stream runs through the same neighbourhoods. Figure 2b illustrates clinical cases recorded in the study regions and the results obtained from Luiz Rau Stream.

According to the normality test results (Table 2), we observed that the data did not follow a normal distribution, necessitating the use of non-parametric tests.

**Table 2:** Shapiro-Wilk *W* test, normally test between points samplings. Source: by Petry et al. 2016

Local	*n*	Shapiro-Wilk *W*	*p* (normal)
PS1	44	0.4821	2.96E−11
PS2	44	0.3909	2.81E−12
LRS1	44	0.2867	2.53E−13
LRS2	44	0.4691	2.08E−11
Pampa Stream	88	0.4388	1.05E−16
Luiz Rau Stream	88	0.3893	2.19E−17
PF2	23	0.2915	1.46E−09
PF1	18	0.2527	1.06E−08
Vicentina WWTP - Feevale	11	0.6601	0.0001446
Vicentina WWTP - UFRGS	28	0.6557	7.14E−07

Statistical analysis using the Friedman test revealed no significant differences in viral load between point 1 (PS1) and point 2 (PS2) of the Pampa Stream (chi^2^ = 0.09; *p* = 0.796; df = 1) (data not shown).

**Fig. 3:**
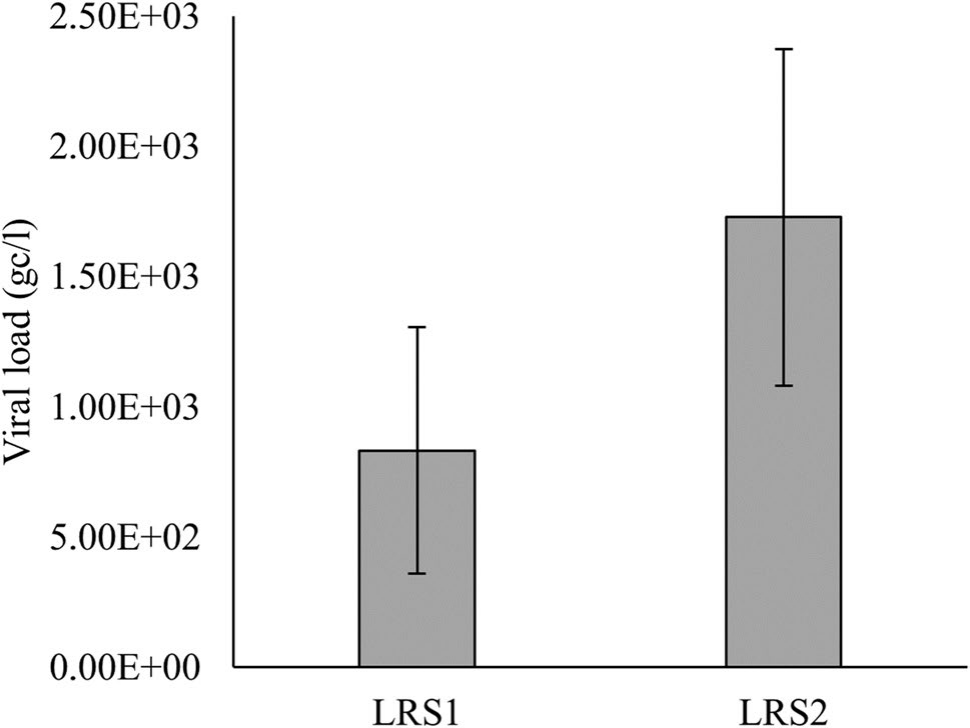
Friedman’s test between points 1 and 2 of Luiz Rau Stream. Source: Petry et al. 2016

Between points one (LRS1) and two (LRS2) of Luiz Rau Stream, the test revealed statistically significant differences in viral load (chi^2^ = 2.75; *p* = 0.029; df = 1) (data not shown). Specifically, LRS2 (mean = 1727.886±646.9) exhibited a higher viral load than LRS1 (mean = 832.668±473.7). These results are depicted graphically in Fig. 3.

We conducted the same test to assess whether there was a statistical difference between the streams, but it indicated no significant variance in viral load values between Pampa and Luiz Rau Streams (chi^2^ = 1.92; *p* = 0.081; df = 1) (data not shown).

Environmental and sample measurements were taken on-site in both streams, revealing no association between viral load values and temperature, indicating that temperature did not impact the detected SARS-CoV-2 RNA viral load in these streams. However, at Vicentina WWTP, an association between environmental and sample temperatures and viral load values was observed (Table 3).

**Table 3:** Spearman test. Source: by Petry et al. 2016

Stream	Temperature	Correlation coefficient of Spearman	*p*-value
Pampa Stream	Environmental	0.082	0.449
Luiz Rau Stream	Environmental	0.122	0.258
Vicentina WWTP	Environmental	0.500	0.006
Dilúvio Stream	Environmental	−0.06	0.758
Pampa Stream	Sample	0.129	0.230
Luiz Rau Stream	Sample	0.112	0.300
Vicentina WWTP	Sample	0.420	0.030
Dilúvio Stream	Sample	−0.288	0.192

**Fig. 4:**
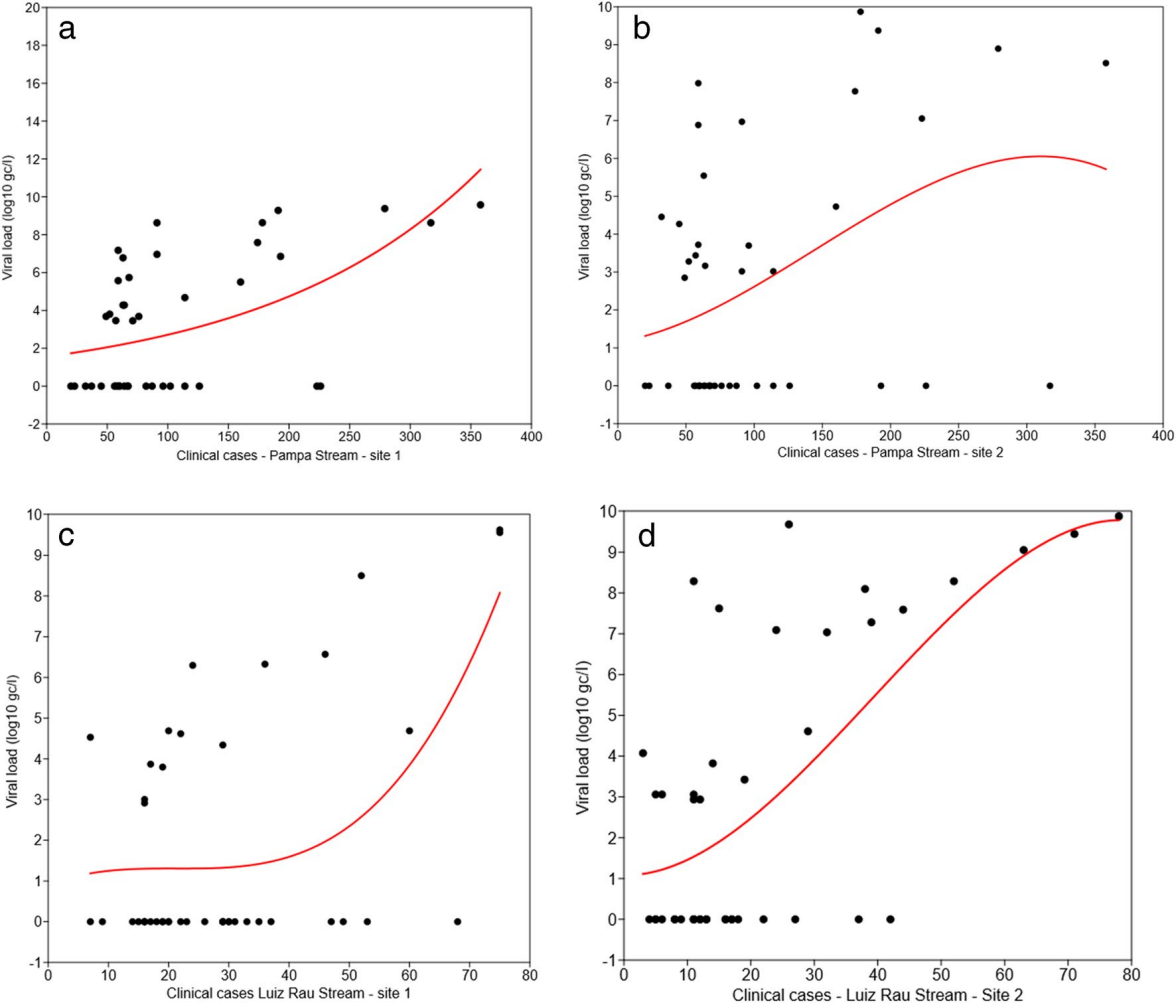
Regression analysis — **a** data fluctuation (*y*-axis) concerning the number of cases (*x*-axis) with an *R*^2^ of 0.61, indicating that the number of cases explains 61% of the variation in the data, while the remaining 39% is attributed to other unmeasured factors; **b** data fluctuation (*y*-axis) related to the number of cases (*x*-axis) with an *R*^2^ of 0.22, indicating that the number of cases explains 22% of the variation in the data, with the remaining variation attributed to unmeasured factors; **c** data fluctuation (*y*-axis) related to the number of cases (*x*-axis) with an *R*^2^ of 0.92, indicating that the number of cases explains 92% of the variation in the data, while the remaining 8% is attributed to other unmeasured factors; **d** data fluctuation (*y*-axis) concerning the number of cases (*x*-axis) with an *R*^2^ of 0.68, indicating that the number of cases explains 68% of the variation in the data, with the remaining 32% attributed to other unmeasured factors. Source: Petry et al. 2016

Regression analysis for both points in Pampa Stream showed statistical significance, indicating that the number of cases influences viral load (PS1: *F* = 21.005; *p* = 2.48E−08; PS2: *F* = 3.8567; *p* = 0.016). With 150 clinical cases, there was a noticeable increase in viral load at point 1 of Pampa Stream (Fig. 4a). In PS2, the trend differed, with a slight increase followed by a decrease in viral loads after 300 clinical cases.

The regression analysis for both points in Luiz Rau Stream was statistically significant, indicating that the number of clinical cases influences the viral load (LRS1: *F* = 169.67; *p* = 28.81E−23; LRS2: *F* = 28.97; *p* = 4.01E−10). In Fig. 4c, we can observe an increase in viral load at point one of Luiz Rau Stream with a progressive ascending curve as the number of cases exceeds 150. Similarly, at point two of Luiz Rau Stream (LRS2), there is a similar trend, showing an increase in viral load from 150 cases onwards, as depicted in Fig. 4d.

In Dilúvio Stream, from 44 samples at each site (22 in DS1 and 22 in DS2), the mean viral load at site one (DS1) was 3.18 × 10^5^ gc/l, and at site two was 6.65 × 10^4^ gc/l. The overall mean at both points was 2.01 × 10^5^ gc/l, with a minimum of 1.27 × 10^4^ gc/l and a maximum of 1.90 × 10^6^ gc/l.

**Fig. 5:**
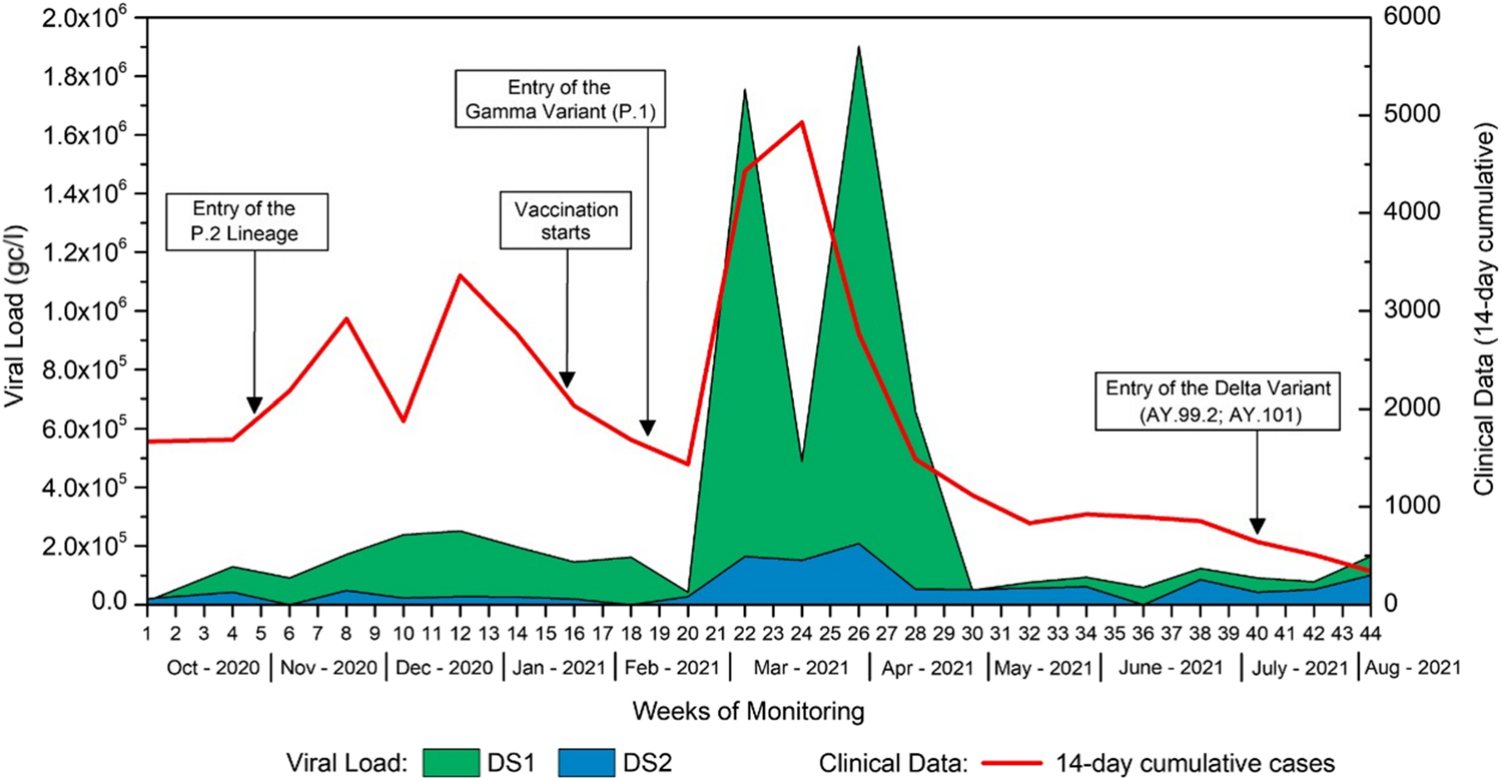
Relationship between clinical cases (14-day moving sum) and VL results in Dilúvio Stream at both points (in gc/l). Source: Petry et al. 2016

Figure 5 shows the same trend between the clinical cases recorded in the study regions and the results of the VL obtained in Dilúvio Stream in Porto Alegre. The graph presents data for clinical cases, considering that this stream runs through multiple neighbourhoods that were included in the sample.

**Fig. 6:**
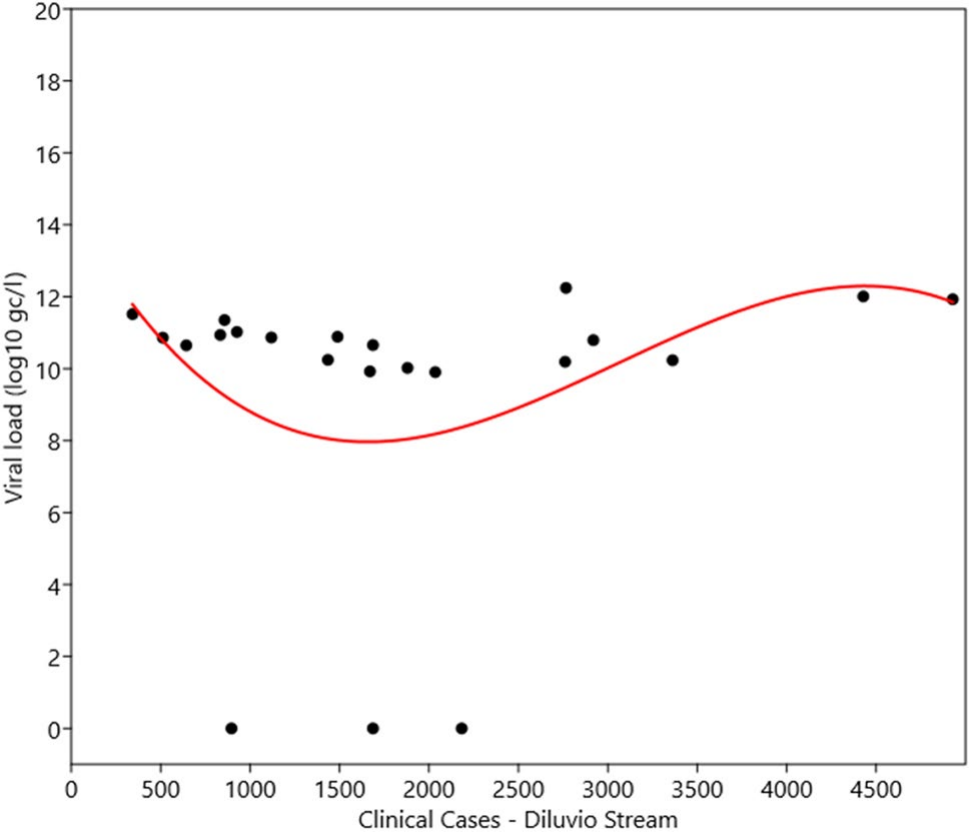
Regression analysis depicting data fluctuations (*y*-axis) with respect to the number of cases (*x*-axis). Source: Petry et al. 2016

In the graph (Fig. 6), we can observe a sudden drop between 1000 and 2000 cases and, from then on, a progressive increase.

**Fig. 7:**
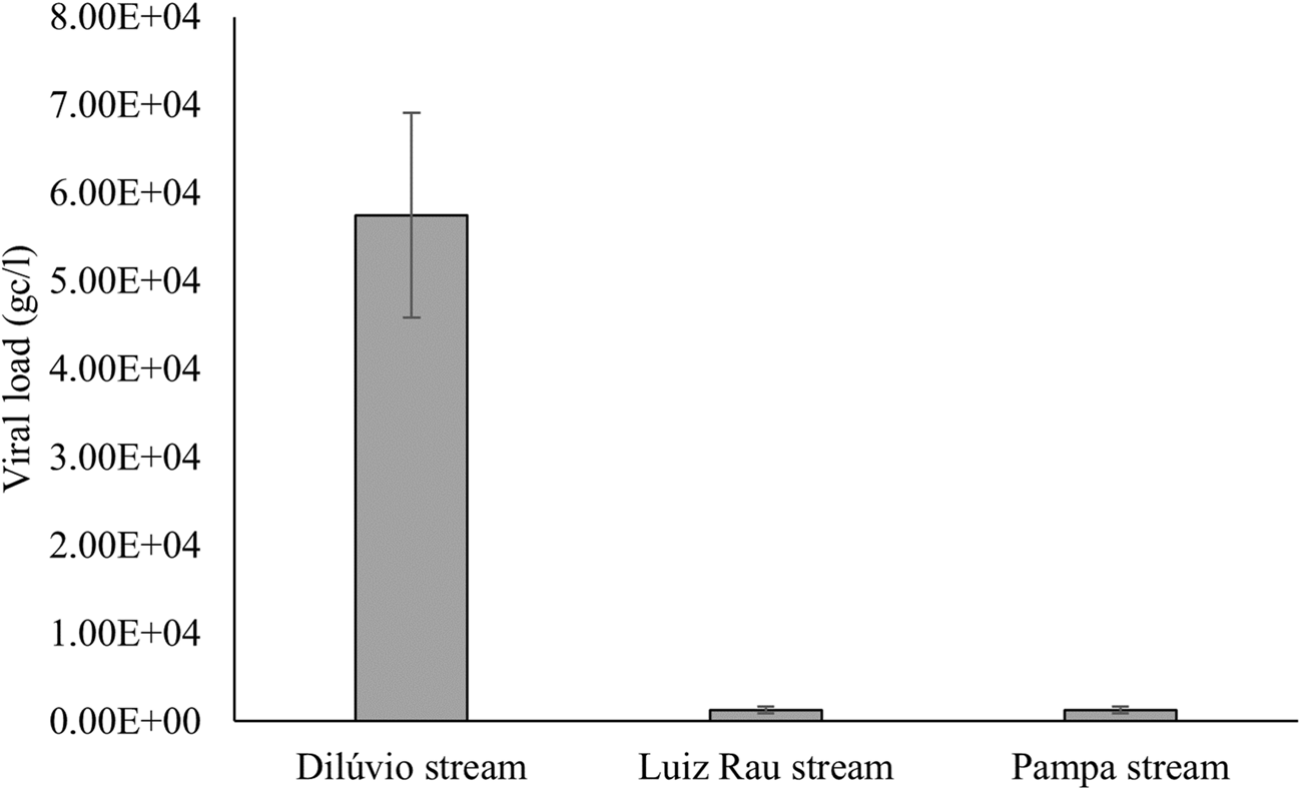
Mann-Whitney pairwise tests between Dilúvio Stream, Luiz Rau Stream, and Pampa Stream. The test indicated statistical differences in viral load among the three streams studied (chi^2^ = 38.29; *p* = 2.86E−10), with the Dilúvio Stream exhibiting a significantly higher viral load than the others. Source: Petry et al. 2016

While there was no statistical difference between the Pampa and Luiz Rau Streams, a notable disparity in viral load orders of magnitude was observed when comparing them to the Dilúvio Stream. This difference is evident not only in the graphs but also in Fig. 7.

### SARS-CoV-2 RNA in the public water fountain

Regarding the public water fountains, 7% (3/41) tested positive for SARS-CoV-2 RNA, with a mean load of 5.02 × 10^1^ gc/l (range: 2.41~8.59 × 10^1^ gc/l).

The statistical test showed no differences between the values of the Canudos and Centro public water fountains (*U* = 200.5; *p* = 0.855) (data not shown).

### SARS-CoV-2 RNA in sewage

**Fig. 8:**
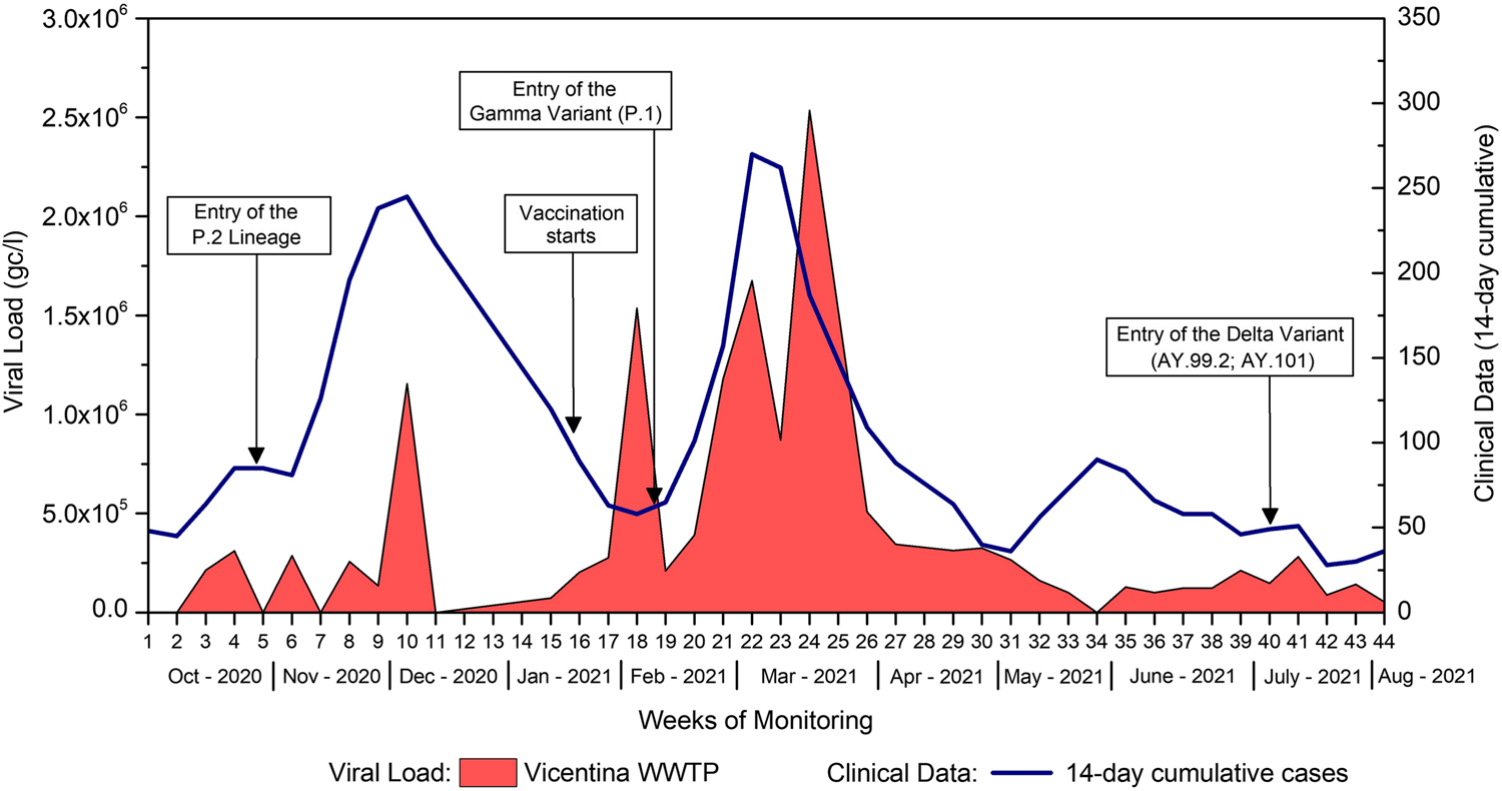
Relationship between the clinical cases (14-day moving sum) and the viral load results in Vicentina WWTP (in gc/l). Source: Petry et al. 2016

In Vicentina WWTP, out of 39 samples collected, the mean viral load was 4.46 × 10^5^ gc/l, ranging from a minimum of 5.42 × 10^4^ gc/l to a maximum of 2.54 × 10^6^ gc/l. Figure 8 illustrates the distribution between the clinical cases recorded in the study regions and the corresponding results obtained in Vicentina WWTP.

**Fig. 9:**
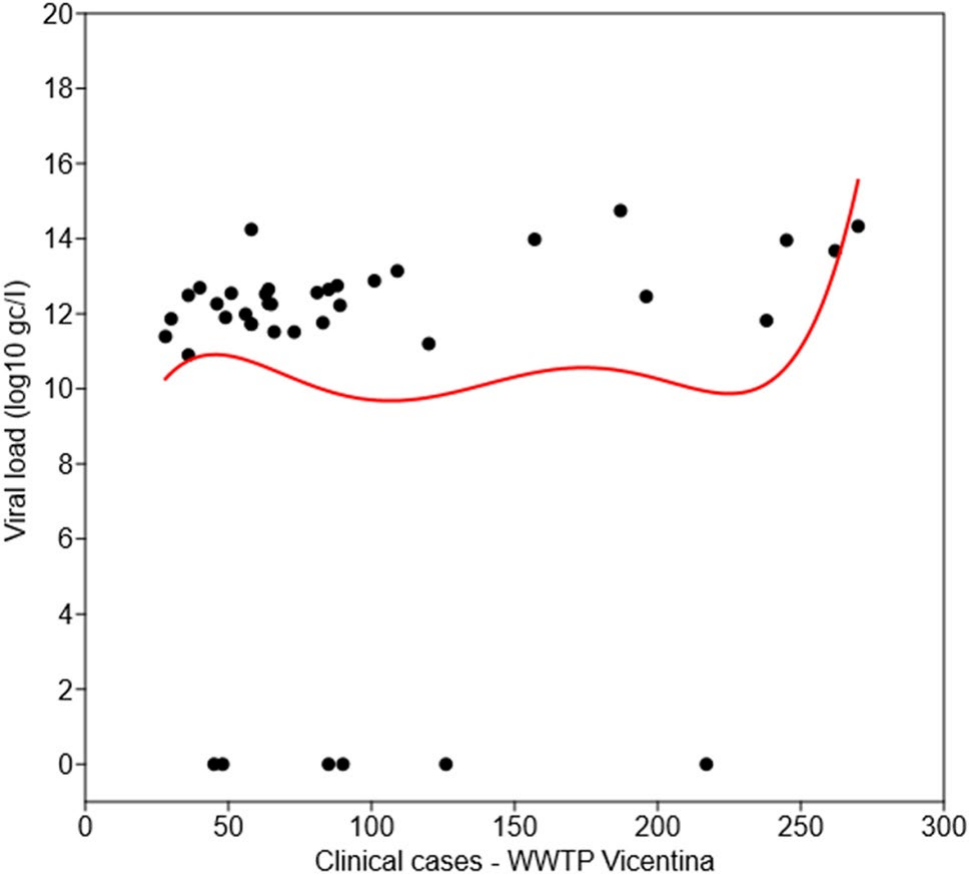
Regression analysis, fluctuation of the data (*y*-axis) with the number of cases (*x*-axis). Source: Petry et al. 2016

In the graph (Fig. 9), we can observe that from 150 cases onwards, there is an increase in the load on the Vicentina WWTP, with a sharp drop between 200 and 250 cases and a new increase after 250 clinical cases.

**Fig. 10:**
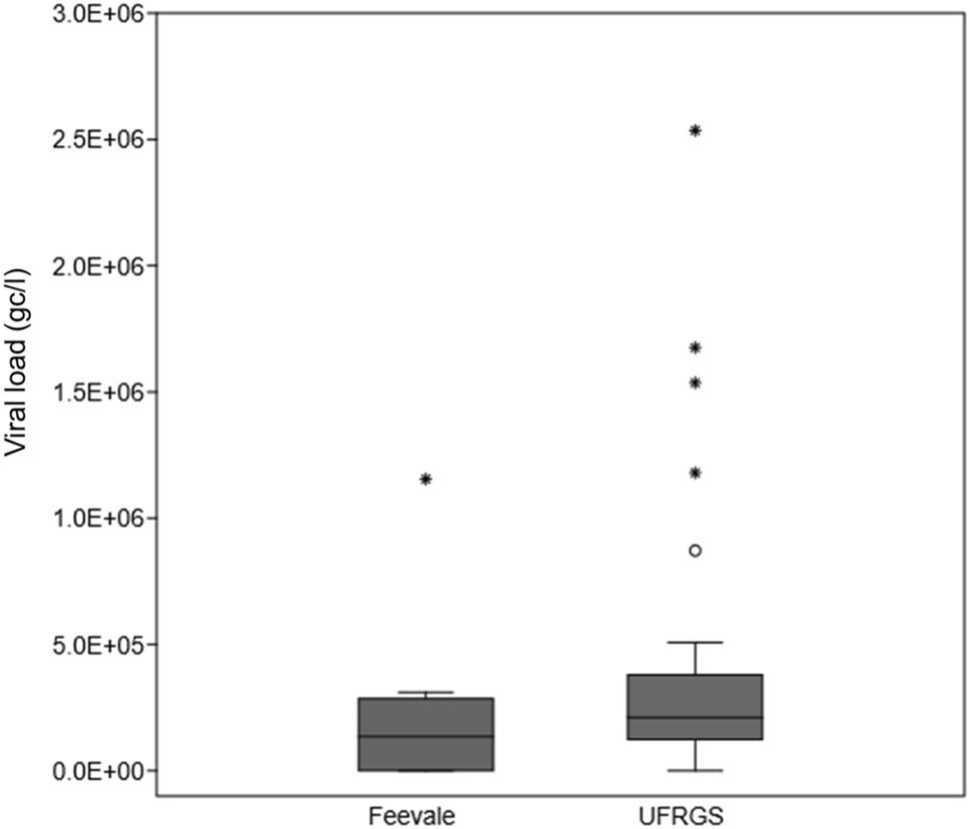
Mann-Whitney test was conducted to compare the methodologies applied by Feevale University and UFRGS in Vicentina WWTP. The test showed no statistically significant difference in viral load between the two methodologies, despite their differences (*U* = 101.5; *p* = 0.105). Source: Petry et al. 2016

Figure 10 shows that despite the universities employing different analysis methods, there were no observed differences in the results at Vicentina WWTP. Feevale University conducted the initial analyses, which were later reviewed by UFRGS.

## Discussion

This is the first study in South Brazil to quantify and compare the presence of SARS-CoV-2 RNA fragments in various environments, including surface freshwater bodies, fountain water, and sewage treatment plants in the state of RS. In another preliminary study on the Dilúvio Stream (FEPAM 2021), environmental monitoring of SARS-CoV-2 was conducted, but only at one point (river mouth). Although it is a composite sample, it is important to monitor different points within the same stream and environments with similar conditions. This allows for the comparison of results between different streams and environments, including surface water, groundwater, and sewage.

The municipalities studied belong to a highly populated metropolitan region with low coverage of the collection and treatment of domestic sewage. The metropolitan region of Porto Alegre also has a high number of confirmed COVID-19 cases; regional and state reference hospitals receive numerous patients, including those from other regions of Rio Grande do Sul (RS) and other Brazilian states. The low sewage treatment rate, population density, and socio-environmental factors were the criteria guiding the monitoring of the streams in these areas since most of the domestic sewage generated in the metropolitan region is discharged directly into these streams.

In Brazil, according to data from the SNIS (2020), only 50.8% of the total sewage generated is treated, and of the sewage collected (IN016), 79.8% is treated. Novo Hamburgo falls significantly below the national average, collecting and treating only 7.17% of the total sewage generated in the city. In Porto Alegre, 64% is collected, of which 81.91% is treated (SNIS 2020). These percentages reflect the precarious housing conditions primarily in the outskirts of these cities, where socially vulnerable populations lack access to drinking water and treated sewage. In some local marginal houses, pipes can be seen directly discharging sanitary effluents into the streams.

The constant flooding of the streams during rainy periods may also lead to the mixing of river water with sewage from septic tanks because these houses are located below the flood level. When the waters recede, all kinds of residues are carried along. These streams are impacted either by direct sewage discharge or indirectly by seasonal flooding.

Due to their precarious housing conditions, the low-income riverside population is usually more susceptible to diseases. Based on our personal observations, we believe that in addition to waterborne diseases, these people have been exposed to respiratory diseases, especially during the COVID-19 pandemic, due to their inability to maintain adequate personal hygiene. During sample collection in both streams and public fountains, it was common to see people circulating without masks, especially in areas with greater socio-environmental vulnerability.

Martins et al. (2022) observed higher rates of cumulative incidence, mortality, and lethality from COVID-19 in neighbourhoods with lower per capita income, highlighting the disproportionate impact of pandemics on socially disadvantaged population groups. The United Nations (UN 2022) declared that COVID-19 has been affecting the Least Developed Countries (LDCs), Landlocked Developing Countries (LLDCs), and Small Island Developing States (SIDS) in unprecedented, profound, and disproportionate ways. These countries, often unable to afford comprehensive response plans, require sufficient support from the international community.

While COVID-19 primarily presents with respiratory symptoms, some patients also report vomiting and diarrhoea. Based on this, studies were conducted in various countries to assess the potential pre-existing presence of SARS-CoV-2 RNA in sewage collected before the onset of the COVID-19 pandemic (Chavarria-Miró et al. 2021; Medema et al. 2020). Other similar research (Bivins et al. 2020; de Oliveira et al. 2021; Sala-Comorera et al. 2021) demonstrated that SARS-CoV-2 virus particles remain viable at 20–24 °C in river water samples (with a T90 of 1.9–2.3 days) and sewage (with a T90 of 1.2–1.6 days).

The environmental monitoring of SARS-CoV-2 in the metropolitan region of Porto Alegre is part of a larger SARS-CoV-2 monitoring programme in the state of RS. In this study, no potentially infectious viruses were detected in samples analysed in a preliminary study conducted by FIOCRUZ (CEVS/SES 2021), which is consistent with the findings of Rimoldi et al. (2020a), where SARS-CoV-2 RNA (orf1ab, N, and E genes) was found in sewage and rivers in the Milan metropolitan region in Italy.

The present monitoring study found no correlation between viral load (VL) and environmental parameters or variations in sample temperature in these streams. However, in the Vicentina WWTP, interference from environmental parameters and sample temperature was observed in the VL. SARS-CoV-2 survival in water depends on several factors, including temperature (coronaviruses are highly temperature-sensitive), light exposure (solar or UV inactivation), the presence of organic matter (viruses can adhere to organic matter particles, affecting settling behaviour or light-shielding), and the presence of antagonist microorganisms (which can increase the extent of inactivation) (Naddeo and Liu 2020), unlike other studies (Bivins et al. 2020; de Oliveira et al. 2021; Jeddi et al. 2022; Kitajima et al. 2020).

The chemical substances present in the sewage may also have interfered with the results, but studies measuring other physicochemical parameters are still needed to confirm this. Unlike our study on surface water, where there was no interference from temperature (both environmental and sample temperature), statistical data from other studies on wastewater and sewage have shown a relationship between results and environmental and sample temperature. This underscores the importance of monitoring surface waters, even though they are highly impacted by domestic sewage, as they are quite distinct environments from sewage systems.

Regression analysis for the Vicentina WWTP yielded statistically significant results, indicating that viral load is influenced by the number of clinical cases (*F* = 5.04; *p* = 0.001). The fifth-order polynomial regression model produced an *R*^2^ of 0.43, suggesting that clinical cases account for 43% of the variation in the data. The remaining percentage can be attributed to other unexplored factors in this study. Alterations in pH, turbidity, suspended solids, the influx of domestic and industrial wastewater, and variations in conductivity, dissolved oxygen levels, and flow rate all potentially impact viral load in sewage. The presence of substances like chlorine and other disinfectants in sewage can further contribute to inhibitory activity, setting sewage apart from what might be found in streams (Figueiredo Costa et al. 2021; Wu et al. 2021).

In our study, we observed that the viral loads in the streams reached averages as high as those in the sewage from Vicentina WWTP. It is worth noting that the domestic sewage discharged into these streams is diluted by the flowing water. While the impacts are significant, these are still natural waters.

Unfortunately, the last water collections from the Centro Water Fountain were impossible to perform due to the absence of water. This is a worrisome situation, especially considering that this water likely comes from a very shallow water table, which is characteristic of intermittent sources and makes them more susceptible to contamination, either from domestic sewage or rainwater. It is also important to emphasize the possibility of cross-contamination due to the intermittence of this collection point.

Although there was a low percentage of positive samples among the other samples evaluated, with one at the Centro Water Public Fountain and two at the Canudos Water Public Fountain, it is important to note that the water from these fountains is used for human consumption. In addition to inadequate plumbing, there is no prior treatment or the necessary water quality control to guarantee its safety for human consumption.

Comparatively, the public water fountains monitored in this study (PF1 and PF2) did not yield significantly different results. However, the positive results coincided with the second and third waves of COVID-19 during the pandemic, which is consistent with a study in the Monterrey metropolitan area, Mexico, conducted by Mahlknecht et al. (2021) on groundwater. This suggests that some untreated sewage may have percolated into the groundwater. In the region where the public water fountains of Novo Hamburgo are located, there may also be some cross-contamination of the waters from these fountains with domestic sewage, as the dates of the samples with positive results coincide with an increase in the number of clinical cases in the region.

Previous studies (Rosiles-González et al. 2019) conducted on groundwater from cenotes have demonstrated the presence of enteric viruses such as human adenovirus and norovirus. The Centro public fountain point in the study carried out in Novo Hamburgo was chosen precisely because it had shown contamination by *E. coli* in a previous collection, as per data provided by the Health Surveillance of the Municipal Health Department of Novo Hamburgo (2020).

The average viral load between Novo Hamburgo streams (Luiz Rau and Pampa Stream) was similar, as confirmed by statistical analysis and observed at two points on the Pampa Stream. On the other hand, there was a significant difference between the two points on the Luiz Rau Stream. The need for collecting and treating domestic sewage along both streams is similar. There are several points where sewage discharge directly into these streams is visibly apparent. It is important to note that points PS1 and LRS1 are less densely populated and still have remnants of vegetation characteristic of riparian forests, besides being closer to the Sinos River mouth. What catches our attention is that the Pampa Stream exhibits characteristics of socio-environmental vulnerability greater than the Luiz Rau Stream. However, there is likely to be as much sewage discharged from the homes of high-income earners as from the homes of low-income earners.

In the case of PS1, the low *R*^2^ suggests that other factors may have influenced the viral load values. The remaining percentage of variation is likely attributed to unmeasured factors, such as changes in water pH, conductivity, dissolved O_2_, flow rate, turbidity, an increase in suspended solids due to domestic sewage discharge, industrial effluents, and irregular disposal of solid waste (rubbish) into the stream. In PS2, the *R*^2^ value at this point was low, and it is possible that other unmeasured or unobserved factors influenced the reduction in the viral load. These factors may include excessive rainfall, which can increase the stream’s water level and dilute domestic sewage discharged at this location, or irregular discharges of industrial effluents that could act as inhibitors of analytical processes.

For the Dilúvio Stream, the regression analysis was statistically significant, showing that viral load is influenced by the number of clinical cases (*F* = 4.42; *p* = 0.016). The third-order polynomial regression model yielded an *R*^2^ of 0.42, indicating that clinical cases explain 42% of the variation in the data. The remaining percentage may be attributed to unmeasured factors, including changes in water pH, conductivity, dissolved O_2_, flow rate, turbidity, an increase in suspended solids due to domestic sewage discharge, industrial effluents, and irregular disposal of solid waste (rubbish) into the stream.

Monitoring water samples from a heavily contaminated stream passing through an urban, underprivileged community without sewage collection in São Paulo (SP State, Brazil) (Pepe Razzolini et al. 2021) showed a statistically significant correlation between cases of COVID-19 and SARS in the community. The authors stated their belief that these results could provide important information for surveillance and controlling the spread in areas with vulnerable populations and poor sanitation.

In our study, the mean viral loads in the streams remained high for much of the monitoring, with the maximum VL corresponding to the second wave of the COVID-19 pandemic, as was also demonstrated in river investigations on the Danube River in Serbia (Kolarević et al. 2021) and in Quito, Peru (Guerrero-Latorre et al. 2020b).

Salvato et al. (2021) detected, for the first time in the State of RS, the P.1 variant in a clinical sample obtained on the 1st of February 2021 from a resident of Gramado, a popular tourist destination in RS, who, however, had no history of travel or contact with travellers returning from northern Brazil. This variant was prevalent in RS from January to March 2021, coinciding with the peaks of reported clinical cases, hospitalizations, and deaths, as demonstrated by da Silva et al. (2021), characterizing the third and largest pandemic wave recorded to date.

The data presented in the environmental monitoring of Dilúvio, Pampa, and Luiz Rau Streams and public water fountains are related to the introduction of variants into the State of RS. The highest viral load (VL) found at these sites corresponds to the periods from November to December 2020, coinciding with the entry of VOC P.2 into RS, and from January to April 2021, coinciding with the entry of VOC P.1 into RS. During these periods, many clinical cases were reported in RS due to the second and third waves of the COVID-19 pandemic, respectively. Our study had not yet commenced during the first wave.

## Conclusions

This innovative study, conducted in Brazil’s southernmost state, focused on urban streams and areas with limited sewage treatment, offering valuable insights into the environmental spread of SARS-CoV-2 in less developed regions. It aids in understanding and mitigating its impact on public health.

Key findings include the absence of infectious viruses in samples, fluctuations in viral load in streams linked to COVID-19 cases, and concerns about SARS-CoV-2 RNA in public fountains. These results highlight water safety challenges in areas with inadequate infrastructure and the vulnerability of disadvantaged populations living near contaminated water sources during pandemics. Continuous monitoring of natural water bodies and improved sewage treatment in urban areas are essential.

However, the study has limitations, such as variations in laboratory methodologies, the need for rapid processing, and challenges related to data collection and reporting errors. These limitations may have led to significant underreporting.

In summary, this research enhances our understanding of SARS-CoV-2 transmission dynamics in environmental settings, benefiting public health and environmental management in the region. It is important to note that the study did not find infectious viruses, suggesting a low risk of waterborne transmission of COVID-19 in the examined environments.

### Supplementary information

ESM 1 (DOCX 290 kb)

## Footnotes

1 Surface water with wastewater: domestic sewage discharged *in natura*, diluted with the surface waters of the streams.
