# Supplementary material for: Environmental monitoring of SARS-CoV-2 in the metropolitan area of Porto Alegre, Rio Grande do Sul (RS), Brazil
Source: Environ Sci Pollut Res Int. 2023 Dec 6;31(2):2129–44. doi: 10.1007/s11356-023-31081-8 (PMC10791933; doi:10.1007/s11356-023-31081-8)
Supplement: Supplementary file 1 — (DOCX 290 kb) [file 11356_2023_31081_MOESM1_ESM.docx]

**SUPPLEMENTARY MATERIAL**

**1 STUDY AREA**

The areas chosen for the present study are in different municipalities, although with similar achievements in terms of sanitary conditions. The micro-basin of the Dilúvio Stream occupies a total area of 83.74 km², 69.50 km² of which belong to Porto Alegre and 14.24 km² to Viamão. The streams waters flow over a length of 17.6 km from its sources to its mouth in the Guaíba Lake, and, of this total, 13.8 km remain in Porto Alegre, crossing the city from east to west, of which about 12 km are channelled. According to Porto Alegre City Hall's diagnosis, the stream runs through several neighbourhoods with high occupation rates. It is the natural receptor of the wastewater of more than 35% of Porto Alegre's total population in its drainage area (about 450 thousand inhabitants). Despite the significant percentage of sewage collection systems implemented in this basin, where only 29.86% of the streets do not have a sewage collection system, the quality of the water of the Dilúvio Stream is highly impaired due to the large contribution of organic load that is discharged into this stream. This fact is related to the irregular connections of domestic sewage in the pluvial network and the state of conservation of the existing pipes in this basin (Prefeitura Municipal de Porto Alegre, 2012). In addition, the stream annually receives about 50,000 cubic meters of soil and garbage in its waters (Ávila et al., 2015).

One of the main tributaries in the lower stretch of the Sinos River is the Luiz Rau Stream, located in the municipality of Novo Hamburgo in the Sinos River Basin. Along its 14 km length, this stream receives an intense discharge of domestic and industrial sewage, called the "Black stream", due to the colour of its waters (FEPAM, 2015). The Water Quality Index (IQA) of the Luiz Rau stream has been consistently given the 'bad' concept throughout the 20-year historical sequence by the State Foundation for Environmental Protection Henrique Luiz Roessler (FEPAM), the agency responsible for monitoring, enforcement, and environmental licensing in RS (FEPAM, 2015). The Pampa Stream receives mainly domestic sewage discharge without any treatment from a region with approximately 40% of the total population of the municipality of Novo Hamburgo. This stream has approximately 9 km in length (do Nascimento & Naime, 2009). It flows into the Sinos River about 1.5 km from the point of collection of raw water for treatment and subsequent public distribution in the city (Monticelli et al., 2017). Vicentina WWTP is in the Vicentina neighbourhood of São Leopoldo and, together with the other WWTP in the municipality, has a coverage index of 41.26% of treated sewage, with the capacity to treat up to 150 litters of sewage per second (SEMAE, 2020).

**Table S1.** Identification, distribution and location of the sampling points and data on frequency, type, and volume and start and end of collection of the sample. Source: by authors (2022).

| **Collection point** | **Code** | **Geographic coordinates** | **Sample volume** | **Start of collection** | **End of collection** | **Type of collection** | **Collection period** | **Samples collected** |
| --- | --- | --- | --- | --- | --- | --- | --- | --- |
| Luiz Rau Stream  Point 1 | LRS1 | 29°43'42.77"S,  51° 7'38.30"W | 500 ml | 01/10/2020 | 02/08/2021 | Grab sample | Weekly | 44 |
| Luiz Rau Stream  Point 2 | LRS2 | 29°42'0.98"S,  51° 8'5.43"W | 500 ml | 01/10/2020 | 02/08/2021 | Grab sample | Weekly | 44 |
| Pampa Stream  Point 1 | PS1 | 29°42'4.83"S,  51° 5'13.95"W | 500 ml | 01/10/2020 | 02/08/2021 | Grab sample | Weekly | 44 |
| Pampa Stream  Point 2 | PS2 | 29°41'24.10"S,  51° 5'10.13"W | 500 ml | 01/10/2020 | 02/08/2021 | Grab sample | Weekly | 44 |
| Dilúvio Stream  Point 1 | DS1 | 30°02'51.6"S,  51°13'46.3"W | 1000 ml | 01/10/2020 | 02/08/2021 | 4-hour composite sample | Fortnightly | 22 |
| Dilúvio Stream  Point 2 | DS2 | 30° 3'20.64"S,  51°10'0.96"W | 1000 ml | 01/10/2020 | 02/08/2021 | 4-hour composite sample | Fortnightly | 22 |
| Centro Water Public Fountain | PF1 | 29°41'3.53"S,  51° 7'34.96"W | 500 ml | 16/09/2020 | 02/08/2021 | Grab sample | Fortnightly | 18 |
| Canudos Water Public Fountain | PF2 | 29°41'10.49"S,  51° 4'36.21"W | 500 ml | 16/09/2020 | 02/08/2021 | Grab sample | Fortnightly | 23 |
| Vicentina WWTP | WWTP | 29°46'26.88"S,  51° 9'24.56"W | 1000 ml | 23/09/2020 | 03/08/2021 | 6-hour composite sample | Fortnightly | 39 |
| **Total** |  |  |  |  |  |  |  | **300** |
